# Analysis of differentially expressed genes in individuals with noninfectious uveitis based on data in the gene expression omnibus database

**DOI:** 10.1097/MD.0000000000031082

**Published:** 2022-10-14

**Authors:** Dandan Zhang, Ning Zhang, Yan Wang, Qian Zhang, Jiadi Wang, Jing Yao

**Affiliations:** a Dalian Women and Children’s Medical Group, China; b The Second Affiliated Hospital of Heilongjiang University of Chinese Medicine, China; c Heilongjiang University of Chinese Medicine, China; d Dalian Port Hospital; e The First Affiliated Hospital of Heilongjiang University of Chinese Medicine, China.

**Keywords:** differentially expressed genes, GEO database, noninfectious uveitis, Protein-protein interaction

## Abstract

Noninfectious uveitis (NIU), an intraocular inflammation caused by immune-mediated reactions to eye antigens, is associated with systemic rheumatism and several autoimmune diseases. However, the mechanisms underlying the pathogenesis of uveitis are poorly understood. Therefore, we aimed to identify differentially expressed genes (DEGs) in individuals with NIU and to explore its etiologies using bioinformatics tools.

GSE66936 and GSE18781 datasets from the gene expression omnibus (GEO) database were merged and analyzed. Functional enrichment analysis was performed, and protein-protein interaction (PPI) networks were constructed.

A total of 89 DEGs were identified. Gene ontology (GO) enrichment analysis identified 21 enriched gene sets. Kyoto encyclopedia of genes and genomes (KEGG) pathway enrichment analysis identified four core enriched pathways: antigen processing and expression signaling, natural killer (NK) cell-mediated cytotoxicity signaling, glutathione metabolic signal transduction, and arachidonic acid metabolism pathways. PPI network analysis revealed an active component-target network with 40 nodes and 132 edges, as well as several hub genes, including *CD27, LTF, NCR3, SLC4A1, CD69, KLRB1, KIR2DL3, KIR3DL1*, and *GZMK*.

The eight potential hub genes may be associated with the risk of developing NIU. NK cell-mediated cytotoxicity signaling might be the key molecular mechanism in the occurrence and development of NIU. Our study provided new insights on NIU, its genetics, molecular pathogenesis and new therapeutic targets.

## 1. Introduction

Uveitis is a vision-threatening eye disease^[1]^ that occurs mostly in people over the age of 36 years.^[2]^ Approximately 10% of blindness in Western countries is caused by uveitis, and 25% of legal blindness in developing countries is associated with uveitis.^[3–5]^ Uveitis cases can be classified into infectious or noninfectious categories based on the etiology.^[6]^ Non-infectious uveitis (NIU) is an intraocular inflammation caused by immune-mediated reactions to eye antigens. Approximately 41% to 55% of the cases are non-infectious uveitis and are associated with systemic rheumatism and several autoimmune diseases^[6,7]^ such as ankylosing spondylitis (AS), Behcet disease (BD), Vogt–Koyanagi–Harada (VKH), and multiple sclerosis.^[8,9]^ Different diseases result in the expression of different proteins.^[10]^ Epidemiologically, uveitis exhibits geographical variations, with NIU being the leading cause of uveitis in industrialized countries affecting 121 in 100,000 people.^[11,12]^ Higher incidences have been observed in some regional groups of the Chinese population,^[13,14]^ but national epidemiological statistics are lacking. An epidemiological survey of uveitis in 8952 people over 40 years of age in Hengli Town, Dongguan City, Guangdong Province, China, suggested that the prevalence of uveitis was 904 per 100,000 individuals, and NIU accounting for 81.5% of the cases.^[15]^ The clinical manifestations of NIU include red eyes, pain, and blurred vision. Vision loss often occurs if the lesions involve the choroid, retina, and macula. These manifestations are characterized by rapid onset, long course, easy recurrence, and many complications, and the blindness rate is higher than in infectious uveitis.^[3]^

The pathogenesis of uveitis is not yet fully understood, and disruption to the stability of the ocular immune microenvironment may be acritical mechanism associated with NIU.^[16]^ Previous studies have revealed that interleukins (IL-17, IL-6, and IL-8) and cytokines (interferon-gamma [IFN-γ] and chemokines) play vital roles in the pathogenesis of intraocular inflammation.^[17–19]^ Some immune-related pathways such as the interleukin (IL)-23/IL-17 pathway, reactive oxygen species signaling pathway, arachidonic acid signaling pathway, and natural killer (NK) cell-mediated cytotoxicity pathway play crucial roles in the pathogenesis of NIU.^[20–24]^ Clear diagnosis and appropriate treatment are essential for preventing irreversible intraocular structural damage and blindness.

Although there are many clinical causes of NIU, the treatment is simple and mainly involves glucocorticoids and/or immune suppressants. In modern medicine, local and systemic corticosteroid therapy is generally used as first-line treatment. Immune modulators such as alkylating agents, antimetabolites, and T-cell inhibitor scan be used to treat patients with poor responses to corticosteroid therapy.^[25]^ Therefore, individualized immunomodulatory treatment programs are required to make the treatment more accurate and effective and to reduce the toxicity and side effects of the drugs.

Recently, biological agents targeting specific molecules have been used to treat NIU. These have attracted attention as alternative therapies for refractory uveitis due to their safety and efficacy.^[26]^ All molecules involved in the immune response, including cytokines, cell adhesion molecules, chemokines, and costimulatory molecules, may be potential targets for biotherapy.^[27]^ Notably, a substantial proportion of the mechanism of protein expression associated with NIU pathogenesis is still lacking and one possible explanation is that unknown genes may partially contribute to the immune deficiency. Therefore, many related genes remain to be identified, these will help us better understand the pathogenesis of NIU and facilitate the discovery of new diagnostic markers or therapeutic targets.

Gene chip technology is a powerful tool for exploring regulatory patterns and molecular mechanisms of targets associated with NIU genesis and development. However, some limitations are associated with current studies based on chip technology. These limitations include the use of small sample sizes and experimental variability in different settings, even when studying the same disease. Integrated bioinformatic techniques are currently employed in biological research. These techniques may be used to identify protein expression and potential mechanisms underlying NIU and provide a strong basis for the precise diagnosis of NIU and the exploration of new drug targets.

In this study, transcriptomic data of patients with NIU and healthy individuals were downloaded from the gene expression omnibus (GEO) database, and multiple microchips were combined. The datasets were used to conduct a preliminary analysis of the potential mechanisms of action underlying the occurrence and development of NIU and provide a basis for experimental research on the pathogenesis of NIU and the identification of its novel therapeutic targets.

## 2. Materials and Methods

### 2.1. Data source

The GEO database (https://www.ncbi.nlm.nih.gov/) was accessed via the national center for biotechnology information website. “Uveitis” was taken as the keyword and used to search for related chip data. Original chip data files labeled GSE66936 and GSE18781 and chip gene annotation files labeled GPL570-55999 were retrieved. The GSE66936 dataset contains 21 samples, including 16from patients with uveitis (experimental group) and 5 from healthy individuals (control group). The GSE18781 dataset contains 55 samples, including 34 from patients with uveitis (experimental group) and 21 from healthy individuals (control group). The 2 sample groups were all patients with NIU. The GSE66936 chip data was generated at NEI Institute in Bethesda, USA, while the GSE18781 chip data was generated at Casey Eye Institute in Portland, USA.

### 2.2. Data collection and analysis of differentially expressed genes (DEGs)

Practical extraction and reporting language script was used to merge data with the same gene names in the GSE66936 and GSE18781 chips, and the Sva package in R software (https://www.r-project.org/, Version4.1.0) was used to correct for merged data in batches. The limma package was used to correct the filtered databased on |LogFC|≥1.5 and *P* < .05, and NIU-related differentially expressed genes were obtained. The heat map package was used to generate volcano plots and heat maps of all the DEGs.

### 2.3. Functional enrichment analysis of DEGs

Cluster Profiler, DOSE, and Enrichplot packages were used to conduct gene ontology (GO) and Kyoto encyclopedia of genes and genomes (KEGG) pathway enrichment analyses of the DEGs. *P* < .05 was used to select enriched gene sets and pathways.

### 2.4. Protein-protein interaction (PPI) network construction and analysis

A PPI network was constructed using the STRING database (http://www.string-db.org). An interaction score >0.4 was used as the threshold. The PPI network was imported into R for visualization, and the connectivity between nodes was calculated to predict the core genes.

### 2.5. Ethics and dissemination

Ethical approval is not required for this differentially expressed gene analysis as we did not use data related to individual patient. The final report of this paper will be published in a peer-reviewed scientific journal or at confer ences to provide evidence-based medical support on NIU, its genetics, molecular pathogenesis and new therapeutic targets for clinical workers, and dataset will be made freely available.

## 3. Results

### 3.1. Identification of DEGs

**Figure 1.**
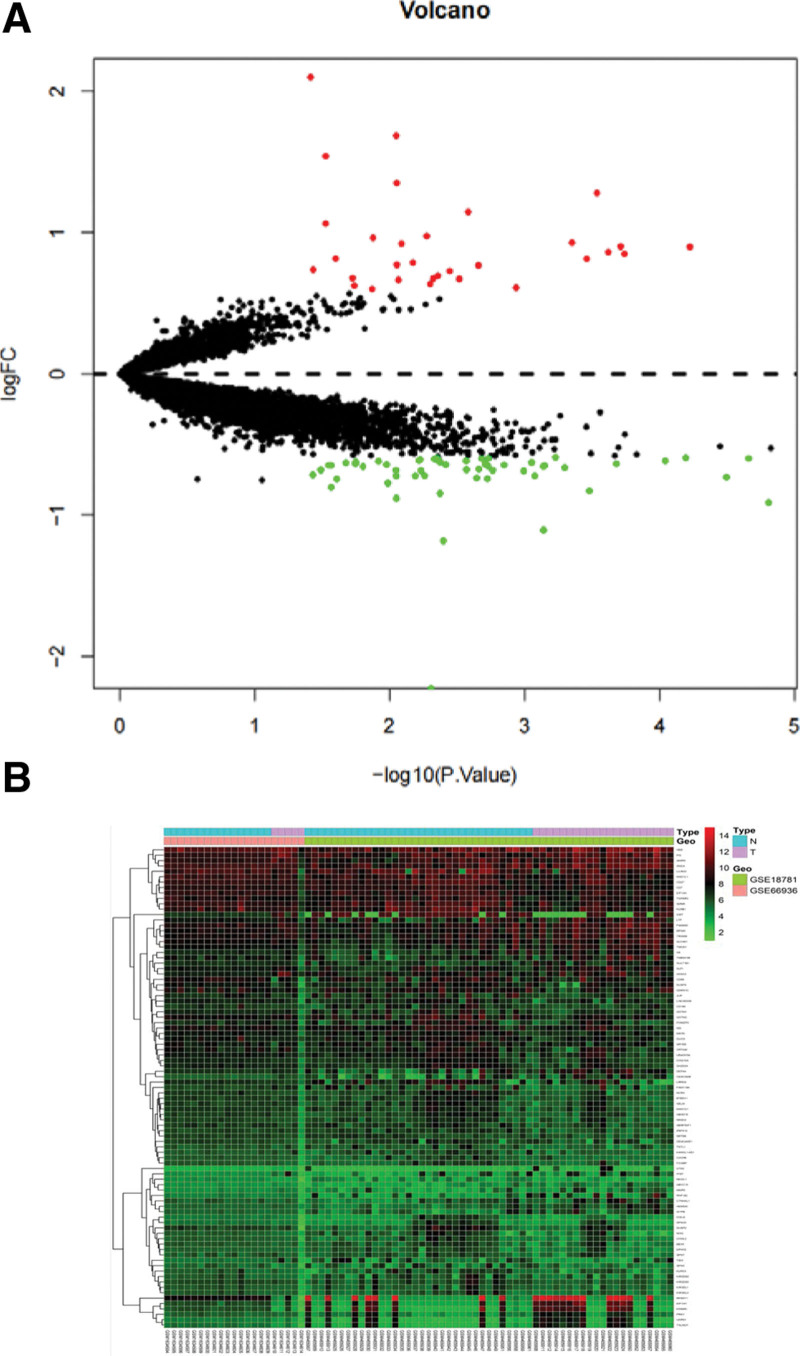
Visualization of differentially expressed genes (DEGs). (A) Volcano map, the vertical coordinate represents the function of gene enrichment, the horizontal coordinate represents the ratio of the number of differential genes in gene enrichment to the total number of differential genes, and different colors represent the *P* value of the significance of enrichment. (B) Heat map, the vertical coordinate represents the function of gene enrichment, the horizontal coordinate represents the ratio of the number of differentially expressed genes to the total number of differentially expressed genes in the function of gene enrichment work set, and the different colors represent the *P* value of enrichment significance.

Analysis of GEO chip data identified genetic variations between healthy individuals and patients with uveitis (|LogFC|≥1.5 and *P* < .05) after batch correction for 89 different genes, including 31 upregulated genes (Table 1) and 58 downregulated genes (Table 2). R software was used to create a volcano plot (Fig. 1A) and heat map (Fig. 1B) of the DEGs.

**Table 1:** NIU corresponds to up-regulated differential genes.

ID	logFC	t	*P* value	B
HEMGN	0.897971	4.245075	5.96E-05	0.95421
FAM46C	0.849353	3.929624	.000182	0.142926
SLPI	0.900781	3.911032	.000194	0.096235
PI3	0.862023	3.849486	.00024	−0.05738
DEFA4	1.279218	3.792935	.000292	−0.19723
BPGM	0.813999	3.741693	.000347	−0.32284
XK	0.927229	3.666386	.000446	−0.50551
ABCC13	0.60886	3.372417	.001161	−1.19525
ANXA3	0.767119	3.165726	.002205	−1.65642
CEACAM8	1.145892	3.107911	.002626	−1.78169
TRIM58	0.671613	3.057149	.003057	−1.89028
MMP8	0.728421	3.002289	.003596	−2.00615
SLC4A1	0.694066	2.933387	.004397	−2.14946
GMPR	0.674664	2.906066	.004758	−2.20558
SULT1B1	0.636764	2.887372	.005021	−2.24376
LTF	0.974396	2.866734	.005326	−2.28568
UTS2	0.787218	2.783662	.006736	−2.4521
IFI27	0.921488	2.713577	.008184	−2.58952
TMOD1	0.664582	2.694538	.008623	−2.62638
GYPB	0.771405	2.684869	.008854	−2.64502
USP9Y	1.349328	2.684208	.00887	−2.64629
EIF1AY	1.68577	2.680707	.008956	−2.65302
RNF182	0.962246	2.53357	.013289	−2.92966
SNCA	0.600163	2.527753	.013494	−2.94034
HBD	0.623051	2.408884	.018352	−3.15411
CTNNAL1	0.676558	2.397402	.018896	−3.17431
PRKY	0.815908	2.282359	.025185	−3.3722
KDM5D	1.539848	2.212397	.029855	−3.48851
TXLNGY	1.062751	2.211743	.029902	−3.48958
TMEM158	0.737262	2.121928	.037008	−3.6343
RPS4Y1	2.099179	2.103238	.038659	−3.66377

**Table 2:** NIU corresponds to down-regulated differential genes.

ID	logFC	t	*P* value	B
NCR3	−0.90988	−4.60725	1.56E-05	1.925831
CD7	−0.5967	−4.51718	2.19E-05	1.680537
ZNF514	−0.73108	−4.41521	3.20E-05	1.405688
SETD6	−0.59294	−4.22519	6.40E-05	0.902048
MATK	−0.61627	−4.12682	9.11E-05	0.645975
NKX3-1	−0.63583	−3.88983	.000209	0.043158
GZMK	−0.82873	−3.75514	.000331	−0.28999
COQ10A	−0.66393	−3.62903	.000505	−0.59526
CXCR6	−0.58928	−3.58162	.000591	−0.70829
EIF1AX	−0.65107	−3.52494	.000711	−0.84213
NOG	−1.10609	−3.51936	.000724	−0.85524
CLIC3	−0.65561	−3.51793	.000728	−0.85859
EPHX2	−0.72139	−3.4729	.000842	−0.96381
ABHD15	−0.62585	−3.45785	.000884	−.99877
FCGBP	−0.6273	−3.45537	.000891	−1.00451
SH2D2A	−0.68754	−3.41413	.001016	−1.09972
NR3C2	−0.64623	−3.30731	.001424	−1.34271
GPX7	−0.68275	−3.24494	.001729	−1.48211
KANSL1-AS1	−0.59473	−3.22329	.001849	−1.53006
MAD1L1	−0.65263	−3.21857	.001876	−1.54047
BEX5	−0.74386	−3.21596	.001891	−1.54623
KIR2DS5	−0.64108	−3.20325	.001966	−1.57424
TKTL1	−0.6229	−3.2024	.001971	−1.5761
SERPINF1	−0.59774	−3.18403	.002085	−1.61643
SELM	−0.67417	−3.16808	.002189	−1.65128
LINC00339	−0.73869	−3.15573	.002273	−1.6782
KIR3DL1	−0.67859	−3.09797	.002706	−1.80305
BTBD11	−0.61627	−3.09733	.002711	−1.80442
CD27	−0.61562	−3.09611	.002721	−1.80705
KIR3DL3	−0.64353	−2.97929	.003847	−2.05425
LRRN3	−1.18212	−2.96509	.00401	−2.08383
NR1D2	−0.62313	−2.94767	.004218	−2.11995
KIR2DS2	−0.84653	−2.94571	.004243	−2.12401
MAN1C1	−0.61801	−2.93756	.004344	−2.14084
TGFBR3	−0.59345	−2.93034	.004436	−2.15573
ID3	−0.60108	−2.91322	.004661	−2.19093
XIST	−2.22804	−2.89302	.00494	−2.23224
COLQ	−0.72076	−2.85404	.005522	−2.31135
CHI3L2	−0.68558	−2.83249	.005871	−2.35475
UBASH3A	−0.60699	−2.82738	.005956	−2.36498
CD160	−0.61415	−2.82059	.006072	−2.37859
KLRC3	−0.72178	−2.79804	.00647	−2.42356
DUSP2	−0.88111	−2.68178	.00893	−2.65097
DUSP5	−0.72236	−2.68064	.008957	−2.65315
JUP	−0.68365	−2.67931	.00899	−2.65571
POMZP3	−0.77376	−2.62854	.010318	−2.75253
CEACAM21	−0.64288	−2.62054	.010543	−2.76767
GSTM1	−0.61873	−2.56905	.012099	−2.86411
CRTAM	−0.65701	−2.46739	.015794	−3.04995
GSTM2	−0.62271	−2.41806	.017928	−3.13791
KLRB1	−0.63632	−2.41675	.017988	−3.14022
GPX3	−0.6292	−2.35254	.021159	−3.25244
CD69	−0.74571	−2.28999	.024716	−3.35933
LILRA3	−0.80325	−2.25113	.027183	−3.4245
CDKN1C	−0.64907	−2.25081	.027204	−3.42503
GPA33	−0.64852	−2.2335	.028372	−3.45375
TSIX	−0.68071	−2.1772	.03248	−3.54586
FAM118A	−0.71538	−2.11973	.037199	−3.63778

### 3.2. GO and KEGG enrichment analysis of DEGs

**Figure 2.**
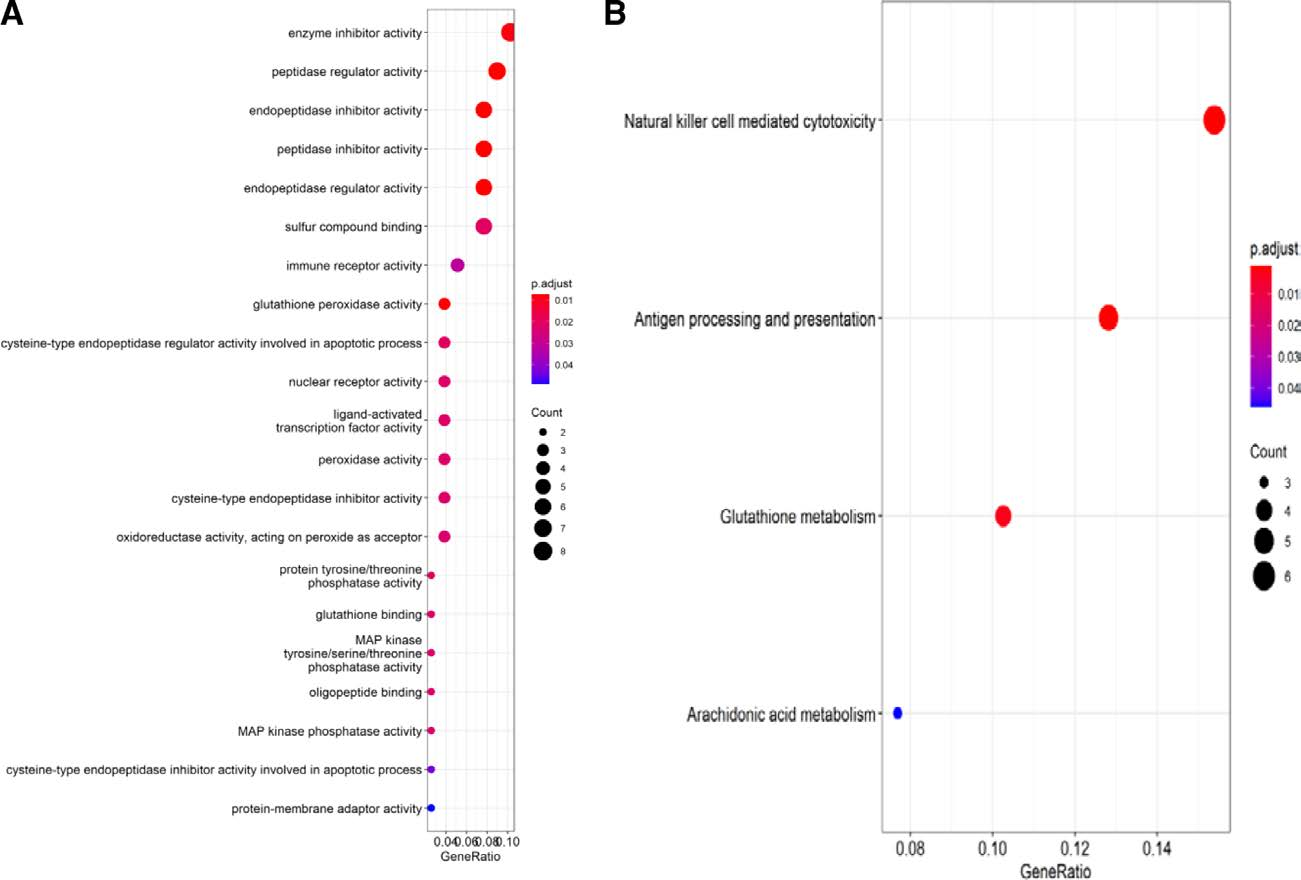
Results of functional enrichment analysis. (A) GO enrichment analysis. (B) KEGG enrichment analysis. KEGG = Kyoto encyclopedia of genes and genomes.

**Figure 3.**
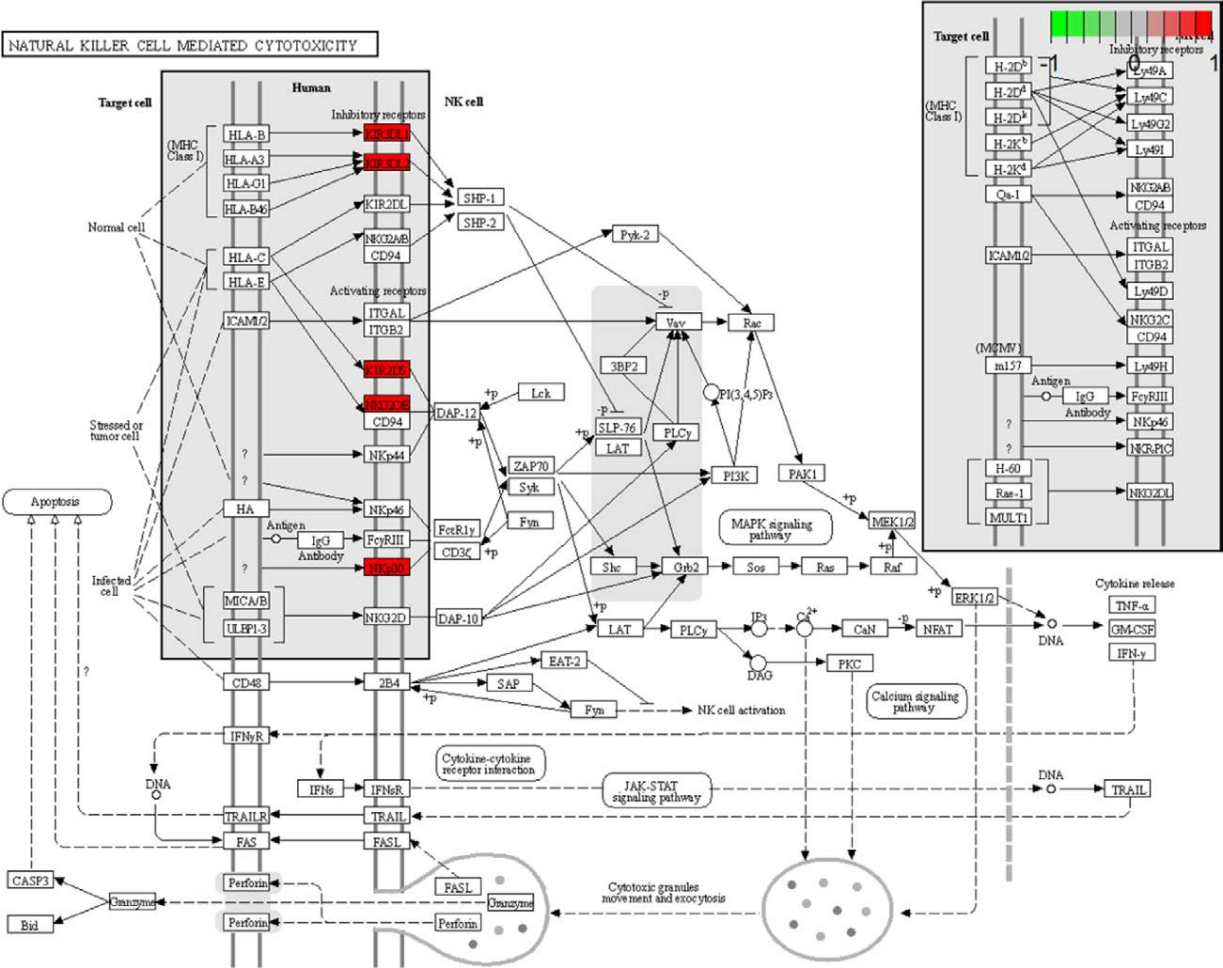
Natural killer cell metabolism mediated cytotoxicity. Core targets are marked in red.

GO enrichment analysis helped identify 21 enriched core gene sets (*P* < .05) (Table 3 and Fig. 2A). KEGG pathway analysis of DEGs identified four enriched pathways (*P* < .05): antigen processing and expression, NK cell-mediated cytotoxicity, glutathione metabolism, and arachidonic acid pathways (Fig. 2B). In these pathways, the ones most closely related to the treatment of NIU were: NK cell-mediated cytotoxicity (Fig.3).

**Table 3:** GO analysis results of DEGs (top 14 terms of BP category were listed). “Count” means how many DEGs are involved.

ID	Description	*p* adjust	Count
GO:0004857	Enzyme inhibitor activity	.008419	8
GO:0061134	Peptidase regulator activity	.007401	7
GO:1901681	Sulfur compound binding	.020612	6
GO:0004866	Endopeptidase inhibitor activity	.007401	6
GO:0030414	Peptidase inhibitor activity	.007401	6
GO:0061135	Endopeptidase regulator activity	.007401	6
GO:0140375	Immune receptor activity	.031426	4
GO:0004869	Cysteine-type endopeptidase inhibitor activity	.022549	3
GO:0016684	Oxidoreductase activity, acting on peroxide as acceptor	.022549	3
GO:0043028	Cysteine-type endopeptidase regulator activity involved in apoptotic process	.020612	3
GO:0004602	Glutathione peroxidase activity	.007401	3
GO:0004879	Nuclear receptor activity	.02193	3
GO:0098531	Ligand-activated transcription factor activity	.02193	3
GO:0004601	Peroxidase activity	.02193	3

### 3.3. PPI network construction and analysis

**Figure 4.**
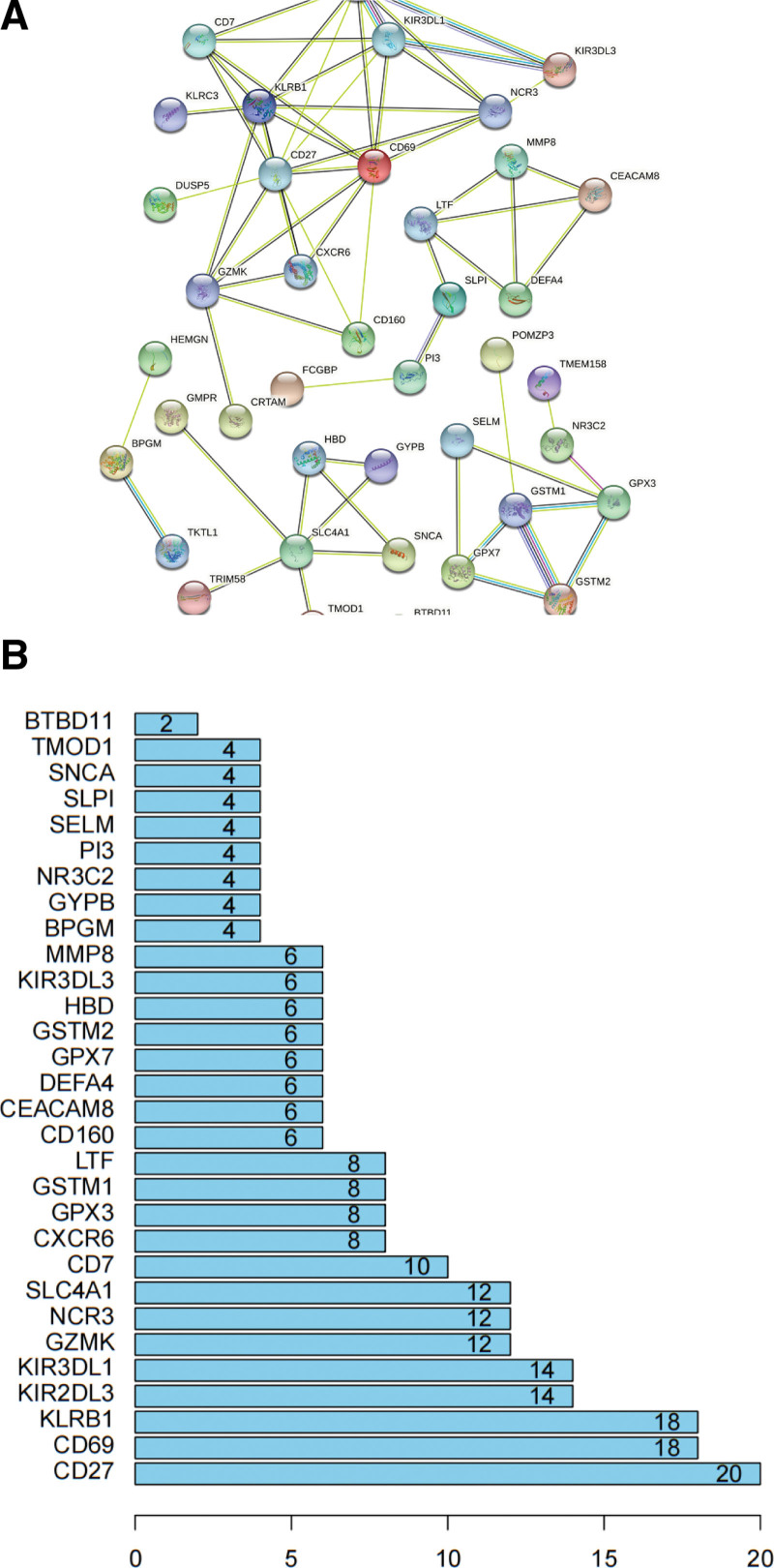
Protein-protein interaction (PPI) network construction analysis.

The network of interactions between proteins encoded by the DEGs was analyzed using the STRING database. The PPI network comprised of 40 nodes and 132 edges (Fig. 4A). Genes with connectivity ≥12 were selected as core genes. The most significant core gene was *CD27*, with connectivity of 20, followed by *CD69, KLRB1, KIR2DL3, KIR3DL1, GZMK, NCR3*, and *SLC4A1* (Fig. 4B).

## 4. Discussion

In this study, we analyzed NIU-related gene expression profiles in GSE66936 and GSE18781 microarray datasets containing 76 samples, including 50 samples from patients with uveitis (experimental group) and 26 samples from healthy individuals (control group). Data in the 2 chips were generated from peripheral blood samples collected from patients with NIU. Bioinformatics analysis-based screening for NIU-related genes helped identify 89 DEGs, including 31 upregulated and 58 downregulated genes. GO enrichment and KEGG pathway analyses revealed that the DEGs were enriched in biological processes such as peptidase regulatory activity, immune receptor activity, and cysteine endopeptidase inhibitor activity. Enriched pathways were mainly involved in antigen processing and presentation, NK cell-mediated cytotoxicity, glutathione metabolism, and arachidonic acid signaling. Among these, peptidase regulatory activity, immune receptor activity, and cysteine endopeptidase inhibitor activity are associated with immunity and inflammation.^[28–30]^ The corresponding genes included *CXCR6, KIR3DL1, CD160, CD27, SNCA, SLPI, NKX3-1, PI3, LTF, SERPINF1*, and *KIR2DS5*. NK cell-mediated cytotoxicity leads to the expression of inhibitory signals on cell surfaces that are associated with uveitis. Immune balance can be achieved by regulating the secretion of cytokines and may contribute to the treatment of uveitis.^[20]^ NK cells have activated and inhibitory receptors on their surface to regulate cell status,^[31]^ CD94/NKG2A is one of the most important inhibitory molecules,^[32]^ that CD94/NKG2A regulates the expression of in CD4 and CD8 + T cells, thereby regulating cytotoxic activity and cytokine production. Clinical studies have demonstrated that combined cyclosporine and prednisone therapy can down-regulate NK-like effector function of CD8 (bright) CD56** + **T cells in patients with NIU.^[33]^ Lipid mediators in the arachidonic acid signaling pathway are closely associated with intraocular inflammation, and drug intervention in the synthesis of lipid mediators may be of great significance in the treatment of uveitis.^[21]^

PPI network analysis of the DEGs performed using the STRING database suggested that *CD27, CD69, KLRB1, KIR2DL3, KIR3DL1, GZMK, NCR3*, and *SLC4A1* were the core genes with the highest connectivity. *CD27*, a member of the tumor necrosis factor receptor superfamily, is a marker of NK cell maturation.^[34]^ *CD27*expression was significantly enhanced in inflamed eye tissue of an experimental autoimmune uveitis model and was associated with uveitis pathogenesis.^[20]^ *CD69* expression in NK gene clusters is a marker of CD4^ + ^T cell activation. Local antigen stimulation triggers CD4^ + ^T cell activation, resulting in the regulation of the immune response through CD69 on the cell surface.^[35]^ *CD69* expression is upregulated during the inflammatory response in BD, AS, VKH, and other immune diseases.^[36,37]^ Killer cell lectin-like receptor B1 (KLRB1; CD161) is expressed in NK cells, CD4^ + ^and CD8^ + ^T cell subsets, and NKT cells^[38]^ and it is correlated with uveitis.^[39]^ Bioinformatics analysis revealed that KLRB1 expression is also significantly upregulated in AS.^[40]^ Therefore, we inferred that high KLRB1 levels during AS might increase the risk of NIU. Paule et al^[41]^ reported that the *CD4/CD8* ratio in the cerebrospinal fluid can be used to infer the etiology of intermediate and/or posterior uveitis, providing novel insights into the role of KLRB1 in NIU pathogenesis. Killer cell immunoglobulin-like receptor 3DL1 (KIR3DL1) and KIR3DL3, members of the killer cell immunoglobulin-like receptor (KIR) family, are NK and T cell subset modulators that protect target cells by inhibiting NK cell activation.^[42,43]^ *KIR3DL1* expression may be involved in the pathogenesis of BD, VKH, and AS.^[44–46]^ *KIR3DL3* is considered a susceptibility gene that is associated with BD. *KIR3DL3* may affect the function of NK cells through genetic differentiation, thus participating in the pathogenesis of BD.^[47]^ *NCR3* encodes NKp30,^[48]^ which can initiate an activation signal cascade in NK cells, leading to cytokine production or cytotoxicity.^[49]^ NK cells are a subgroup of lymphocytes. Kucuksezer et al^[23]^ confirmed that NK cells play a role in experimental autoimmune uveitis and may be a potential pathological factor. Considering that NCR3 is highly expressed in patients with NIU, inhibition of NCR3 activation may play a vital role in the treatment of NIU. *SLC4A1* stimulates the functional activity of innate immune cells and is involved in immune responses, T cell activation, toll-like receptor-binding regulatory pathways, and granulocyte activation, all of which are closely associated with NIU.^[50–53]^ Granzyme K (GZMK) is one of the pro-inflammatory members of the GZM family that can combine with IFN-γ to induce mouse fibroblasts to secrete inflammatory cytokines,^[54]^ enhancing the inflammatory function of non-immune cells.^[55]^ Its abnormal expression is associated with various auto immune diseases, including RA, AS, and scleroderma,^[11]^ that can lead to the development of NIU. Additional studies on GZMK may provide new perspectives on the diagnosis, treatment, and pathogenesis of NIU.

In the current study, we discussed 8 potential crucial genes that are involved in the occurrence and development of NIU. These 8 crucial genes were involved in the activation and regulation of NK cells. NK cell-mediated cytotoxicity was the key pathway enriched in this study, suggesting that these genes may serve as potential biomarkers and therapeutic targets for NIU. However, the limitations of this study should be considered. First, it is difficult to consider some important factors such as region, race, and age. Considering that the etiology of NIU is complex, undetermined factors such as region and history should be evaluated in further studies. In addition, potential key genes need to be further validated by RT-qPCR in clinical samples. Finally, the mechanisms by which these genes work are not fully understood. More evidence is needed to investigate their biological foundation.

## 5. Conclusions

Understanding the cellular and biochemical events involved in NIU is essential for treating the disease. In this study, gene expression data were analyzed to identify core genes involved in NIU and to determine the underlying mechanism associated with the disease. Bioinformatics analyses revealed that the NK cell-mediated cytotoxicity pathway plays a vital role in the pathogenesis of NIU, and genes associated with this pathway included *CD27, CD69, KLRB1, KIR3DL1, KIR3DL3*, and *NCR3*. The results of this study provide novel insights for studying the underlying mechanisms and activation factors associated with NK cells in NIU. In addition, analysis of NIU core genes suggested that NIU is associated with several autoimmune diseases and that different diseases induce the release of diverse proteins. Thus, using bioinformatic techniques to identify the expression of genes and the potential mechanisms underlying NIU may provide a strong basis for its precise diagnosis and exploration of novel drug targets.

## Acknowledgments

We would like to thank Editage (www.editage.cn) for English language editing.

## Author contributions

**Conceptualization:** Jing Yao.

**Data curation:** Ning Zhang, Yan Wang, Qian Zhang, Jiadi Wang, Jing Yao.

**Formal analysis:** Ning Zhang.

**Methodology:** Jiadi Wang.

**Software:** Dandan Zhang, Qian Zhang.

**Writing – original draft:** Dandan Zhang, Ning Zhang, Jing Yao.

**Writing – review & editing:** Dandan Zhang, Ning Zhang.
